# Supplementary material for: Crystal dissolution by particle detachment
Source: Nat Commun. 2023 Oct 9;14:6300. doi: 10.1038/s41467-023-41443-y (PMC10562397; doi:10.1038/s41467-023-41443-y)
Supplement: Supplementary file 1 — Supplementary Information File [file 41467_2023_41443_MOESM1_ESM.pdf]

Supplementary Information for

Crystal Dissolution by Particle Detachment

Guomin Zhu[1,2], Benjamin A. Legg[1], Michel Sassi,[1] Xinran Liang[1], Meirong Zong[1], Kevin M. Rosso[1], and James J. De Yoreo[1,2,*]

1 Physical Sciences Division, Pacific Northwest National Laboratory, Richland, WA 99354, USA.

2 Department of Materials Science and Engineering, University of Washington, Seattle, WA 98195, USA.

* Correspondence to: Email: james.deyoreo@pnnl.gov

	S_p (MeV cm^2 g^{-1})	I (nA)	a (µm)	ψ (Gy s^{-1})
**Low dose**	2.36	0.7	0.3	5.84×10^8
**High dose**	2.36	2.0	0.1	1.67×10^9

Supplementary Table 1. Details of the value used to calculate the dose rate of a 300 kV electron beam.

Species	D (nm^2 s^{-1})
OH•	2.80
H•	7.00
O_2^-	2.10
HO_2	2.00
HO_2^-	2.00
H_2O_2	2.20
e_{aq}^-	4.50

Supplementary Table 2. Diffusion coefficients for the main radiolytic species of interests.

Species	pH 3 high dose		pH 5 high dose		pH 7 high dose		pH 3 low dose		pH 5 low dose		pH 7 low dose	
	$T_{1/2}$ (μs)	λ (μm)	$T_{1/2}$ (μs)	λ (μm)	$T_{1/2}$ (μs)	λ (μm)	$T_{1/2}$ (μs)	λ (μm)	$T_{1/2}$ (μs)	λ (μm)	$T_{1/2}$ (μs)	λ (μm)
OH•	16.35	0.52	11.57	0.44	11.35	0.44	26.49	0.67	20.87	0.59	20.26	0.58
H•	5.30	0.47	2.75	0.34	2.67	0.34	9.41	0.63	5.54	0.48	5.34	0.47
O_2^-	0.57	0.08	2.94	0.19	3.03	0.20	0.60	0.09	4.64	0.24	4.88	0.25
HO_2	5.43	0.26	4.74	0.24	4.70	0.24	7.64	0.30	6.70	0.28	6.61	0.28
HO_2^-	0.12	0.04	0.16	0.04	0.16	0.04	0.13	0.04	0.19	0.05	0.19	0.05
H_2O_2	41.11	0.74	10.77	0.38	10.44	0.37	99.55	1.15	19.23	0.50	18.17	0.49
e_{aq}^-	0.31	0.09	0.60	0.13	0.61	0.13	0.42	0.11	0.98	0.16	1.00	0.16

Supplementary Table 3. Calculated average lifetime ($T_{1/2}$) and diffusion range ($\lambda=\sqrt{6\tau D}$) for the radiolytic species of interest (water + 0.2 mM oxalate).

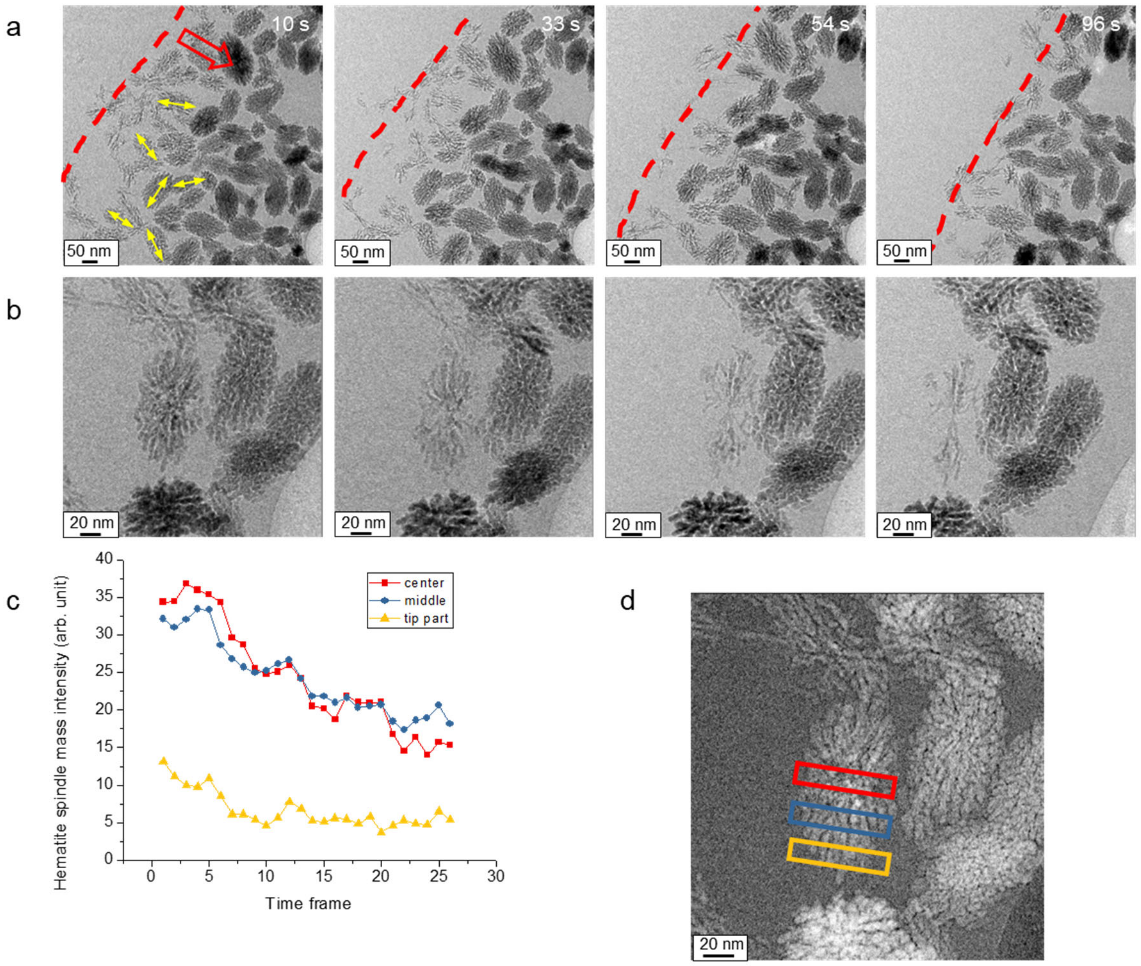

Supplementary Figure 1. Dissolution behavior of spHm. a, Example of spHm dissolution demonstrating the movement of dissolution front drawn as the red dashed line. Similar dissolution behavior is shown independent of the direction of the spindle pointing towards (yellow arrow) b, Another example showing the skeleton formation during the dissolution. c, time dependent mass change of the center, middle and tip part of the spindle as drawn in d. The tip dissolves slower compared to the center and middle. The mass change is calculated according to the intensity change.

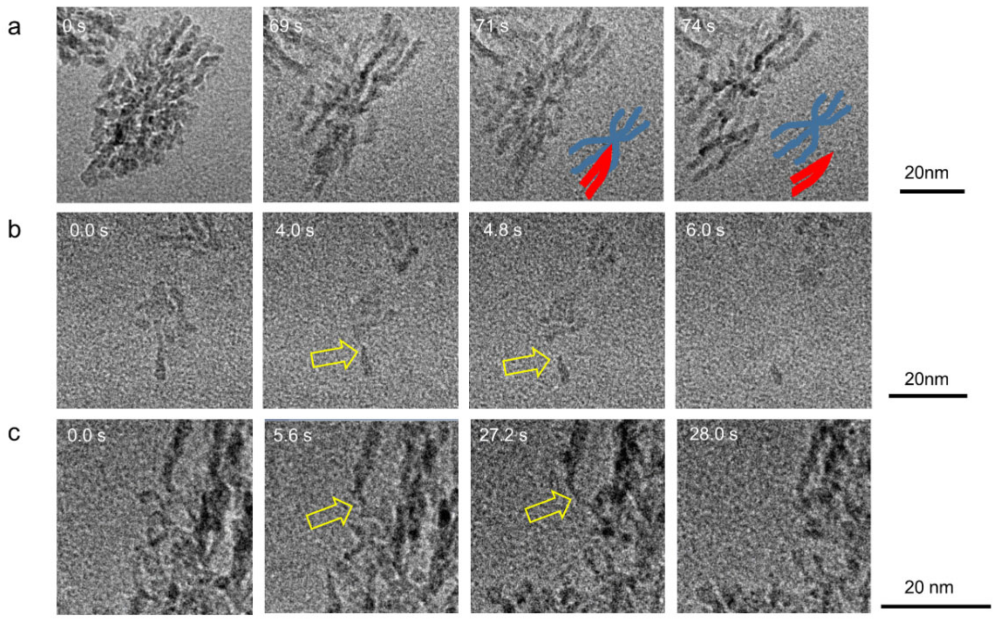

Supplementary Figure 2. More examples of particle detachment and skeleton formation. a, Detachment of a big fragment from 71 s to 74 s (Supplementary Movie 7), illustrated by the enclosed cartoon. b, c, Examples of sequential TEM images of necking and particle detachment, the yellow arrow highlights the necking region (Supplementary Movie 4).

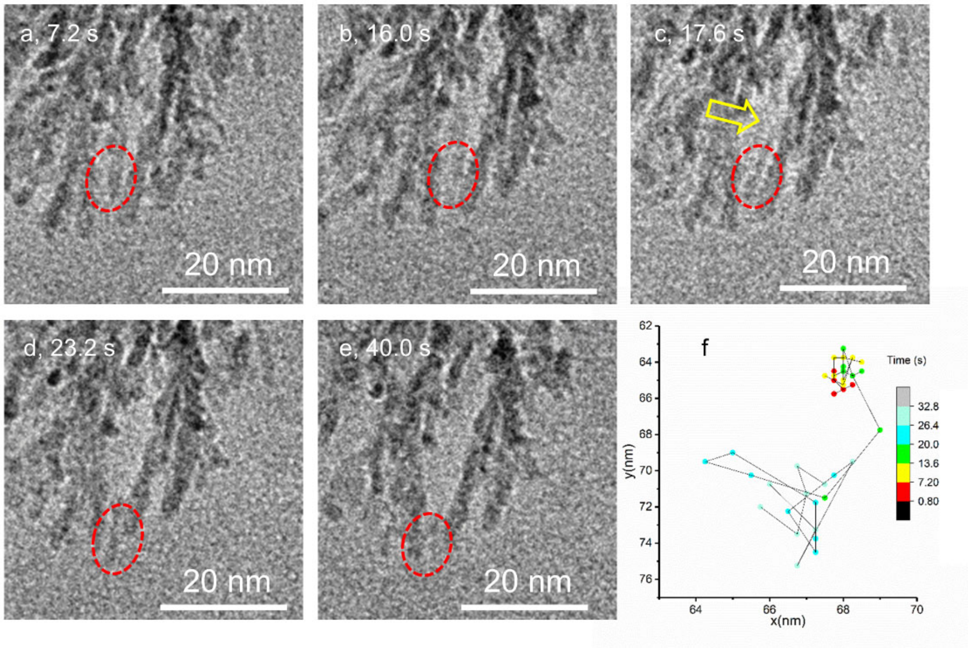

Supplementary Figure 3. Trajectory of a fragment after break-up and detachment from the spindle. a-e, sequential TEM images showing the break-up and detachment. The yellow arrow highlights the place where the structure detached from the spindle. Red circle highlights detached structure at 17.6 s (c), and its trajectory of movement is shown in f.

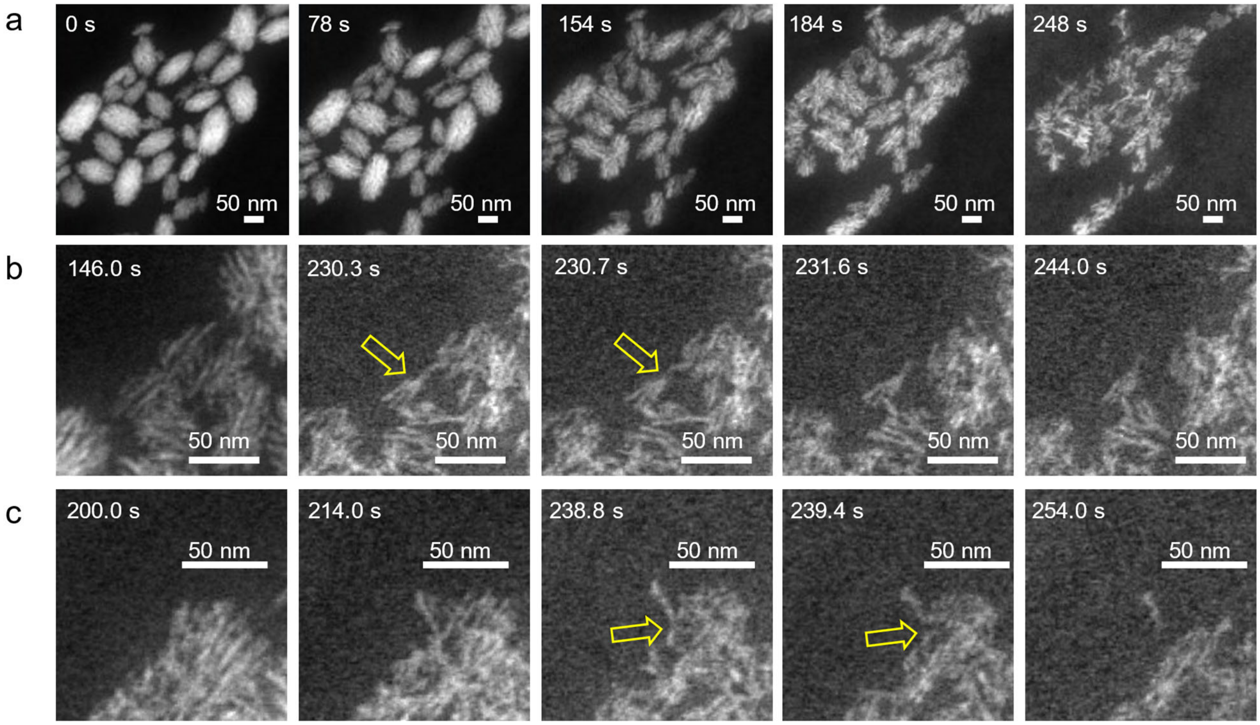

Supplementary Figure 4. Particle detachment and skeleton formation revealed in the STEM mode at different doses. a, Selected time-lapse STEM images extracted from Supplementary Movie 8 showing skeleton formation during dissolution. b, c, Two examples of time-lapse STEM images highlights the process of neck break-up and particle detachment during dissolution. The arrows highlight the area where the neck break-up happens.

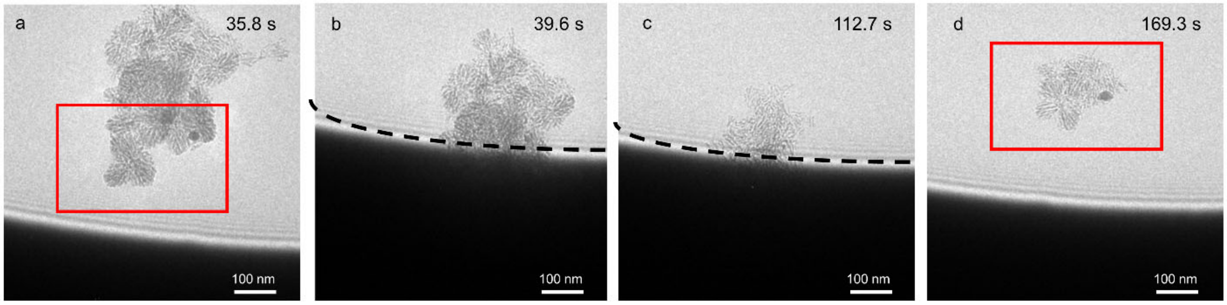

Supplementary Figure 5. Hm dissolution in the electron-blocking area due to diffusive radicals. a, TEM image of aggregates of spHm. b and c, We moved the edge of beam only to irradiate the top part of the aggregates. d, after continuous imaging for a while, we shifted the beam to look at the Hm, which were in the electron-blocking area, as highlighted by the rectangle.

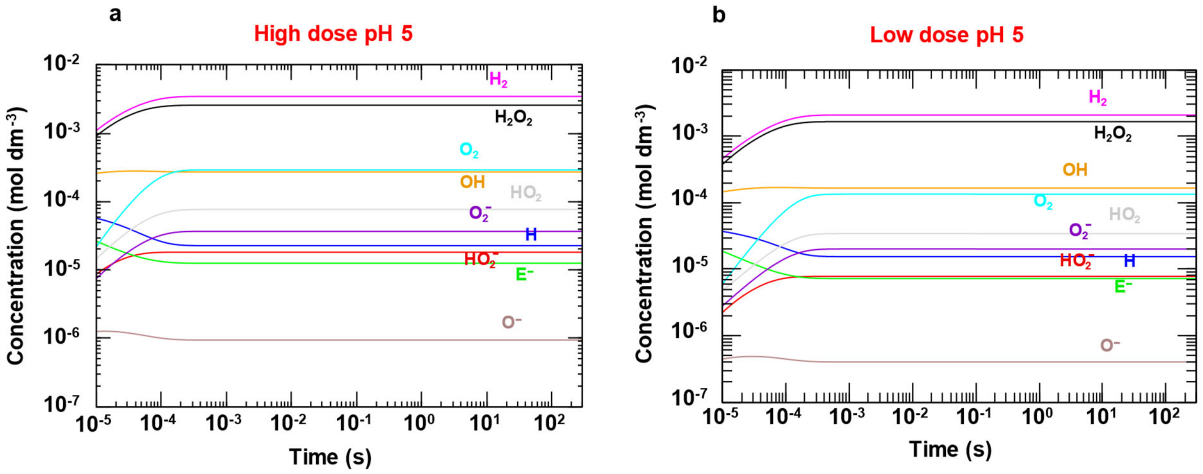

Supplementary Figure 6. Calculation of concentration profile of free radicals in a high dose and low dose condition. High dose rate condition is 2 nA, 0.1 μm beam radius (a), while the low dose condition is 0.7 nA and 0.3 μm (b).

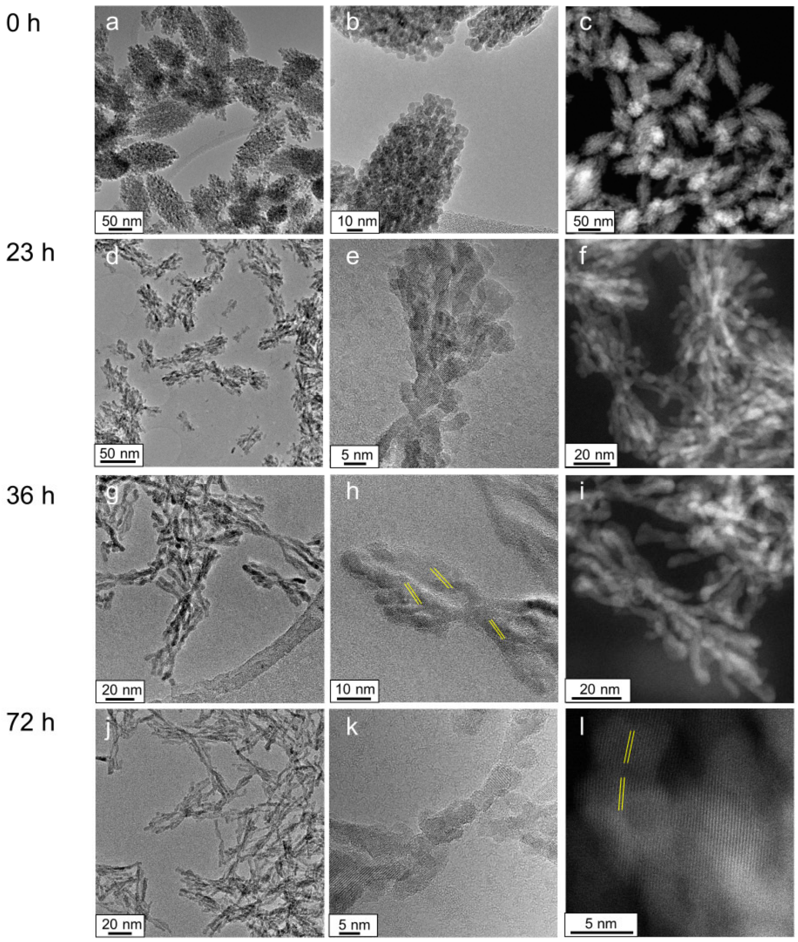

Supplementary Figure 7. TEM/STEM images of the ex-situ dissolution of spHm mesocrystal showing similar skeleton structure evolution compared to the in-situ TEM/STEM dissolution. Typical TEM/STEM images of spHm overtime at 0 h (a-c), 23 h (d-f), 36 h (g-i), and 72 h (j-l). c, f, i, and l are STEM images, while all others are TEM images. TEM and STEM modes were both applied to better probe the structure. We studied the interface within the rod-like structure using both HRTEM (h) and HRSTEM (l). Mismatch was found within the structure as highlighted in h and l.

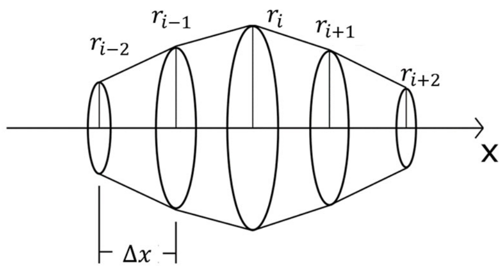

Supplementary Figure 8. Approximate of the rod and neck geometry using conical segments. r_i is the radii of the conical segments and Δx is the spacing.
