# Supplementary material for: Crystal dissolution by particle detachment
Source: Nat Commun. 2023 Oct 9;14:6300. doi: 10.1038/s41467-023-41443-y (PMC10562397; doi:10.1038/s41467-023-41443-y)
Supplement: Supplementary file 3 — Description of Additional Supplementary Files [file 41467_2023_41443_MOESM3_ESM.pdf]

Description of Additional Supplementary Files

Supplementary Movie 1: in situ TEM observation of rhHm dissolution, 10 X, 145 k, 80 e Å^{-2} s^{-1}

Supplementary Movie 2: in situ TEM observation of rhHm dissolution, 10 X, 185 k, 456 e Å^{-2} s^{-1}

Supplementary Movie 3: in situ STEM observation of rhHm dissolution, 20x, 160 k

Supplementary Movie 4: in situ TEM observation of spHm dissolution, 8 x, 115 k, 28 e Å^{-2} s^{-1}

Supplementary Movie 5: in situ TEM observation of spHm dissolution, 1 x, 115 k, 28 e Å^{-2} s^{-1}

Supplementary Movie 6: in situ TEM observation of spHm dissolution, 1 x, 115 k, 28 e Å^{-2} s^{-1}

Supplementary Movie 7: in situ TEM observation of spHm dissolution, 8 x, 115 k, 28 e Å^{-2} s^{-1}

Supplementary Movie 8: in situ STEM observation of spHm dissolution, 10 x, 115 k

Supplementary Movie 9: in situ TEM observation of spHm dissolution, 20 x, 185 k, 456 e Å^{-2} s^{-1}

Supplementary Movie 10: in situ TEM observation of spHm dissolution, 20 x, 69 k, 4 e Å^{-2} s^{-1}

Supplementary Movie 11: in situ TEM observation of mixed spHm and rhHm dissolution, 16 x, 34 k, 1 e Å^{-2} s^{-1}
